# Low prevalence of connexin-40 gene variants in atrial tissues and blood from atrial fibrillation subjects

**DOI:** 10.1186/1471-2350-13-102

**Published:** 2012-11-07

**Authors:** Gregory D Tchou, Robert C Wirka, David R Van Wagoner, John Barnard, Mina K Chung, Jonathan D Smith

**Affiliations:** 1 Department of Cell Biology, 9500 Euclid Ave, Cleveland, OH, 44106, USA; 2 Cleveland Clinic Lerner College of Med of Case Western Reserve University, 9500 Euclid Ave, Cleveland, OH, 44106, USA; 3 Department of Molecular Cardiology, 9500 Euclid Ave, Cleveland, OH, 44106, USA; 4 Department of Cardiovascular Med, 9500 Euclid Ave, Cleveland, OH, 44106, USA; 5 Department of Quantitative Health Sciences, Cleveland Clinic, 9500 Euclid Ave, Cleveland, OH, 44106, USA

**Keywords:** Atrial fibrillation, Connexins, Ion channels, Genetics, Allelic expression imbalance

## Abstract

**Background:**

The atrial gap junction protein connexin-40 (Cx40) has been implicated to play an important role in atrial conduction and development of atrial fibrillation (AF). However, the frequency of Cx40 mutations in AF populations and their impact on Cx40 expression remains unclear. In this study, we sought to identify polymorphisms in the Cx40 gene *GJA5*, investigate the potential functional role of these polymorphisms, and determine their allelic frequencies. The prevalence of nonsynonymous Cx40 mutations in blood and atrial tissue was also compared to mutation frequencies reported in prior studies.

**Methods:**

We conducted direct sequencing of the *GJA5* coding and 3^′^ UTR regions in blood samples from 91 lone AF subjects and 67 atrial tissue-derived samples from a lone cohort, a mixed AF cohort, and several transplant donors. Reporter gene transfection and tissue allelic expression imbalance assays were used to assess the effects of a common insertion/deletion polymorphism on Cx40 mRNA stability and expression.

**Results:**

We identified one novel synonymous SNP in blood-derived DNA from a lone AF subject. In atrial tissue-derived DNA from lone and mixed AF subjects, we observed one novel nonsynonymous SNP, one rare previously reported synonymous SNP, and one novel 3^′^ UTR SNP. A previously noted 25 bp insertion/deletion polymorphism in the 3^′^ UTR was found to be common (minor allele frequency = 0.45) but had no effect on Cx40 mRNA stability and expression. The observed prevalence of nonsynonymous Cx40 mutations in atrial tissues derived from lone AF subjects differed significantly (p = 0.03) from a prior atrial tissue study reporting a high mutation frequency in a group of highly selected young lone AF subjects.

**Conclusions:**

Our results suggest that Cx40 coding SNPs are uncommon in AF populations, although rare mutations in this gene may certainly lead to AF pathogenesis. Furthermore, a common insertion/deletion polymorphism in the Cx40 3^′^ UTR does not appear to play a role in modulating Cx40 mRNA levels.

## Background

Atrial fibrillation (AF) is the most commonly encountered form of sustained cardiac arrhythmia in clinical practice, with a prevalence in the USA that is projected to increase three-fold between the years 2000 and 2050 [1-3]. AF is a complex disease characterized by irregular electrical activity within the atria, resulting in episodes of uncoordinated atrial contraction that substantially increase stroke risk and mortality [4-6]. Studies investigating the genetic basis of the disease have shown that somatic and germ line variants in the atrial gap junction protein connexin-40 (Cx40) can disrupt normal atrial conduction and may predispose individuals to idiopathic, or lone, AF [7-9]. The Cx40 gene *GJA5* encodes two alternative transcripts, *Cx40A* and *Cx40B*, comprised of first exons 1A or 1B and a shared coding second exon, respectively [10]. We previously described rs10465885, a common single nucleotide polymorphism (SNP) in the *Cx40B* promoter that strongly affects *Cx40* mRNA expression and is associated with early onset lone AF [11]. We hypothesized that direct sequencing of the *GJA5* region in atrial and blood DNA from expanded cohorts of mixed and lone AF patients might yield additional polymorphisms altering *Cx40* structure or expression, potentially resulting in a predisposition to AF.

## Methods

### Study subjects

Most (64 of 67) of the atrial appendage tissues were obtained from a biorepository of human atrial tissues from patients undergoing coronary artery bypass grafting, valve surgery and/or the Maze surgical ablation procedure. Subjects had a history of AF and were categorized as “lone AF” if they did not have structural heart disease, or “mixed AF” if they had AF in combination with coronary artery disease or valvular heart disease (mitral regurgitation). The remaining three atrial tissue samples were obtained from non-failing donor hearts not used for transplant with unknown cardiac phenotypes (Table 1). Use of discarded atrial tissues from surgical subjects was approved with an exemption for written consent by the Cleveland Clinic Institutional Review Board and performed in accordance with institutional guidelines. Atrial specimens were obtained from unmatched organ donor hearts, after informed consent by a surviving family member and used under a protocol approved by the Cleveland Clinic Institutional Review Board.

**Table 1:** Patient characteristics

	**Atrial tissues**			**Blood samples**
	**Lone AF**	**Mixed AF**	**Donor**	**lone AF**
	**N=34 (%)**	**N=30 (%)**	**N=3 (%)**	**N=91 (%)**
Age, years, mean ± SD (range)	53.8 ± 9.6	66.3 ± 9.8	50 ± 15.6	57.8 ± 11.5
	(31–71)	(32–86)	(32–60)	(25–84)
Sex, male	28 (82.4)	17 (56.7)	2 (67)	67 (73.6)
Race, Caucasian	32 (94.1)	29 (96.7)	3 (100)	87 (95.6)
Unknown or Other	2 (5.9)	1 (3.3)	0	4 (4.4)
Cardiac Disease			ND	
CAD	0	3 (9.1)		0
Valvular disease	0	21 (63.6)		0
CAD + Valve disease	0	6 (18.2)		0
Unknown	0	0		0
AF Burden			ND	
Paroxysmal	12 (35.3)	6 (20.0)		39 (42.9)
Persistent	6 (17.6)	5 (16.7)		33 (36.3)
Permanent	16 (47.1)	19 (63.3)		13 (14.3)
Unknown	0	0		6 (6.6)
Rhythm at the time of sampling			ND	ND
Sinus	16 (47.1)	10 (33.3)		
AF	18 (52.9)	20 (66.7)		
Unknown	0	0		

AF = atrial fibrillation, CAD = coronary artery disease, ND = not determined.

Blood samples were obtained from the Cleveland Clinic Lone Atrial Fibrillation GeneBank (CCAF), which enrolled subjects at least 18 years of age who had a history of AF without coronary heart disease. Specifically, these subjects had no significant coronary artery disease (<50% coronary artery stenosis if cardiac catheterization was performed or a normal stress test) and normal left ventricular function (left ventricular ejection fraction ≥50%). Patients were excluded if they had structural heart disease or congenital heart disease, except for an isolated patent foramen ovale with normal right heart chambers; significant valvular disease (>2+ regurgitation or any valve stenosis); or prior percutaneous coronary intervention or coronary artery bypass grafting. Patients with hypertension were not excluded. Written informed consent was obtained from all CCAF subjects under a protocol approved by the Cleveland Clinic Institutional Review Board and performed in accordance with institutional guidelines. There were no subjects from whom we obtained both atrial tissue- and blood-derived DNA.

### Preparation of genomic DNA, total RNA and complementary DNA from human atrial tissue and blood

Atrial genomic DNA (gDNA) and total RNA was prepared from atrial tissue samples using Qiagen kits. cDNA was produced with the iScript Select cDNA Synthesis Kit (BioRad) using an oligo(dT)_20_ primer. Buffy coat gDNA was prepared from blood samples of subjects in the CCAF Genebank using the MasterPure DNA Purification Kit for Blood Version II (Epicentre Biotechnologies).

### Firefly luciferase-*GJA5* 3^′^ UTR Expression Vector Construction

The *GJA5* 3^′^ UTR region (1,938 bp) from atrial tissue gDNA was amplified by PCR using the upstream primer ATGAT**CTCGAG**AAGCGACGTCTTAGTAAGGCCAG and the downstream primer ATGCAT**GGGCCC**CCTTTACCCATCCCATCAGCACC (XhoI and ApaI sites in bold). PCR products were cloned into the pGEMT-easy vector (Promega), transformed into DH5α *Escherichia coli*, and screened for the presence of the insert. Wild-type and variant *GJA5* 3^′^ UTR insert sequences were then directionally cloned into a modified pcDNA3-Luc vector, immediately downstream of the luciferase coding region. The resulting constructs were co-transfected into HL-1 murine atrial cardiomyocytes [12] with a β-galactosidase-containing transfection control vector, and the Dual-Light reporter gene assay system (Tropix) was used to measure luciferase and β-galactosidase activities in cell lysates according to methods previously described [11]. The mRNA instability positive control was generated by annealing the single-stranded oligos TCGAC**ATTTATTTATATTTATTTA**CCGCGCGGCGCCG and TCGACGGCGCCGCGCGG**TAAATAAATATAAATAAAT**G, which contain an AU-rich element (ARE, bold emphases) that reduces mRNA stability when present in the 3^′^UTR [13]. Annealed oligos were phosphorylated with T4 polynucleotide kinase (M0201, NEB) and cloned into the XhoI site of the *GJA5* 3^′^ UTR luciferase reporter described above.

### *GJA5* gDNA and cDNA Sequencing

Screening of 67 atrial derived gDNA samples for the 25 bp insertion/deletion polymorphism was carried out using the PCR primers ATTCCTCGGAGTAGTGGTGAGATGG (upstream) and ACACCCTAGCAGAAGGAAAGGTTGC (downstream), which amplify a 131 bp region in the *GJA5* 3^′^ UTR. For sequencing of the *GJA5* coding region in gDNA derived from blood or atrial tissue, two overlapping fragments of *GJA5* were amplified using the primer pairs ACGAGTACCCGGTGGCAGAGAAGGC (upstream) and AGGAGCCAAGCAGTGATGACAGTGAGAA (downstream) and GCTAATATGGCTACTTTGAATCTTCTC (upstream) and ACATGCAGGGTGGTCAGGAA (downstream), respectively. The *GJA5* PCR fragments were sequenced and analyzed for SNPs using the SNP Analysis tool hosted by the Laboratory of Population Genetics, National Cancer Institute, NIH (http://lpg.nci.nih.gov/LPG) [14]. To measure allelic expression imbalance, we used a highly sensitive sequencing technique that compares cDNA and gDNA allelic ratios in heterozygous individuals [15]. This is an adaptation of a similar technique using single base extension that also compares allelic ratios in cDNA vs. gDNA in heterozygous subjects [16,17]. A *Cx40A* fragment containing exon 1A, the coding region and approximately 1 kb of the 3^′^ UTR (including the 25bp insertion/deletion and an indicator SNP rs1043806) was amplified from eight atrial tissue-derived cDNA samples using the PCR primers GGTGGAAGAGGAACAACTGA (upstream) and GGGCCTCCATAGCTGTCATCA (downstream). A portion of the *GJA5* 3^′^ UTR containing the indicator SNP rs1043806 was amplified from gDNA using the PCR primers ATTCCTCGGAGTAGTGGTGAGATGG (upstream) and GGGCCTCCATAGCTGTCATCA (downstream). The *Cx40A* cDNA fragment and the gDNA *GJA5* 3^′^ UTR fragment corresponding to each subject were sequenced using Sanger methodology on an Applied Biosystems 3730xl DNA analyzer. The sequence trace files were analyzed using PeakPicker 2 software (publicly available at *http://genomequebec.mcgill.ca/publications/pastinen/*) [15] to determine the cDNA allelic expression ratio, normalized to the gDNA ratio, at the rs1043806 position as previously described [11].

## Results

### Patient Characteristics

Table 1 shows the demographics of 91 lone AF blood donors and a separate group of 67 subjects who provided atrial tissue. Of the 67 atrial appendage donors, 34 had lone AF (no significant structural heart disease), 30 had AF with coronary artery disease or valvular heart disease (mitral regurgitation), and 3 were derived from heart transplant donors with limited phenotypic information. Approximately half (56.7%) of the 67 tissue donors were in AF at the time of surgery.

### Single nucleotide polymorphisms in the connexin-40 coding region

**Figure 1:**
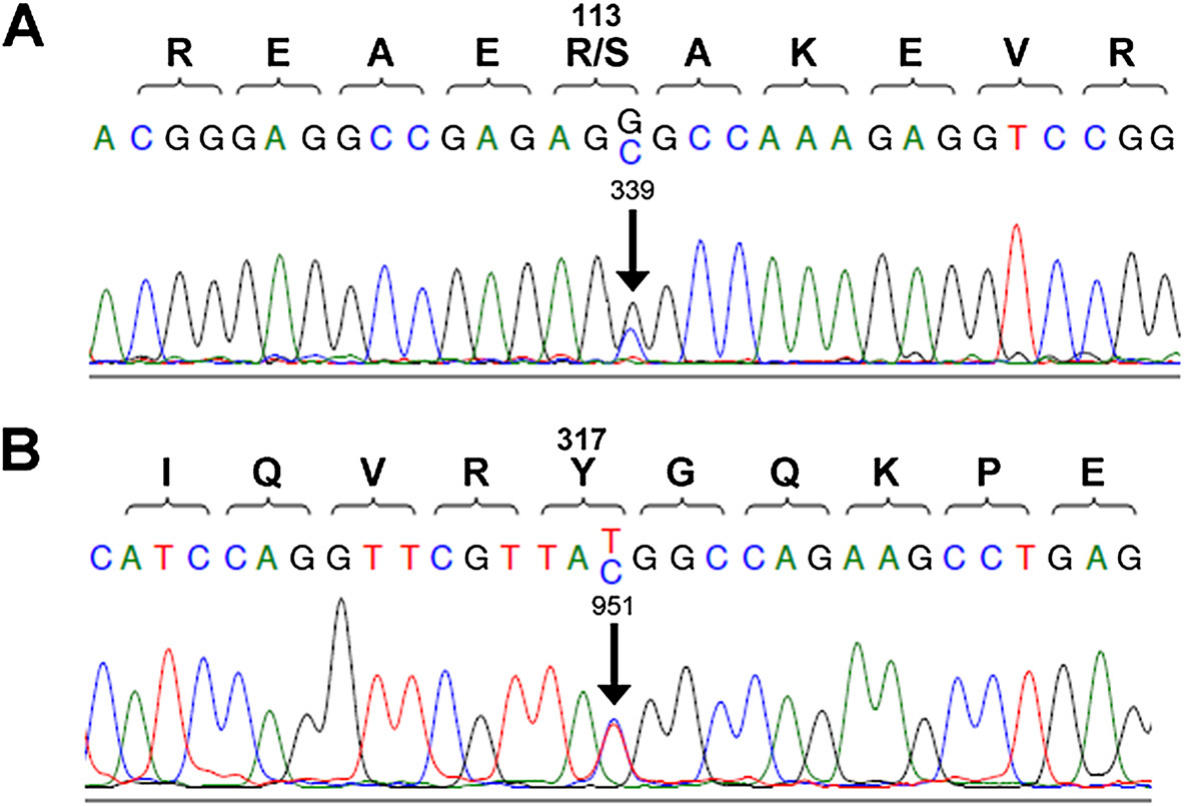
**Sequence traces of novel *Cx40 *SNPs.** (**A**) Sequence of 339G>C, a novel nonsynonymous *Cx40* SNP identified in a lone AF atrial tissue sample. 339G>C results in the cytoplasmic loop substitution R113S. (**B**) Sequence of 951T>C, a novel synonymous *Cx40* SNP identified in a lone AF blood sample.

In order to screen for and assess the frequency of nonsynonymous *Cx40* variants in a large cohort of subjects of European ancestry, we conducted direct sequencing of the *Cx40* coding region in blood- and atrial tissue-derived gDNA samples from patients diagnosed with lone or mixed AF, as well as a small number of transplant donors. Detected SNPs were determined to be novel if they have not been previously reported in public SNP databases, including those available from dbSNP [18] and the Thousand Genomes Project [19]. Out of 34 atrial tissue samples obtained from lone AF patients (Table 1), a single novel nonsynonymous *Cx40* coding SNP was identified (339G>C) (Figure 1A). The novel 339G>C SNP results in a substitution of serine for arginine in the cytoplasmic loop of Cx40 (R113S) at a position that is highly conserved in mammals. Analysis with the PolyPhen-2 tool predicts that R113S is likely to be functionally and structurally well-tolerated [20]. Since blood samples matching our atrial tissue samples were not available, we were unable to determine whether the 339G>C variant had a somatic or germline origin. No novel SNPs were observed through direct sequencing of 33 atrial tissue samples from a mixed AF and donor cohort (Table 1). Sequencing of 91 blood samples from a lone AF cohort (Table 1) yielded one novel synonymous *Cx40* coding SNP (951T>C, Figure 1B) and one previously reported synonymous coding SNP (rs2232191) in separate individuals. A search in dbSNP shows that the latter SNP is rare in the HapMap European and African populations, but has an allele frequency of ~ 5% in the Chinese and Japanese populations [18].

### Identification of variation in the proximal region of the 3^′^ UTR

**Figure 2:**
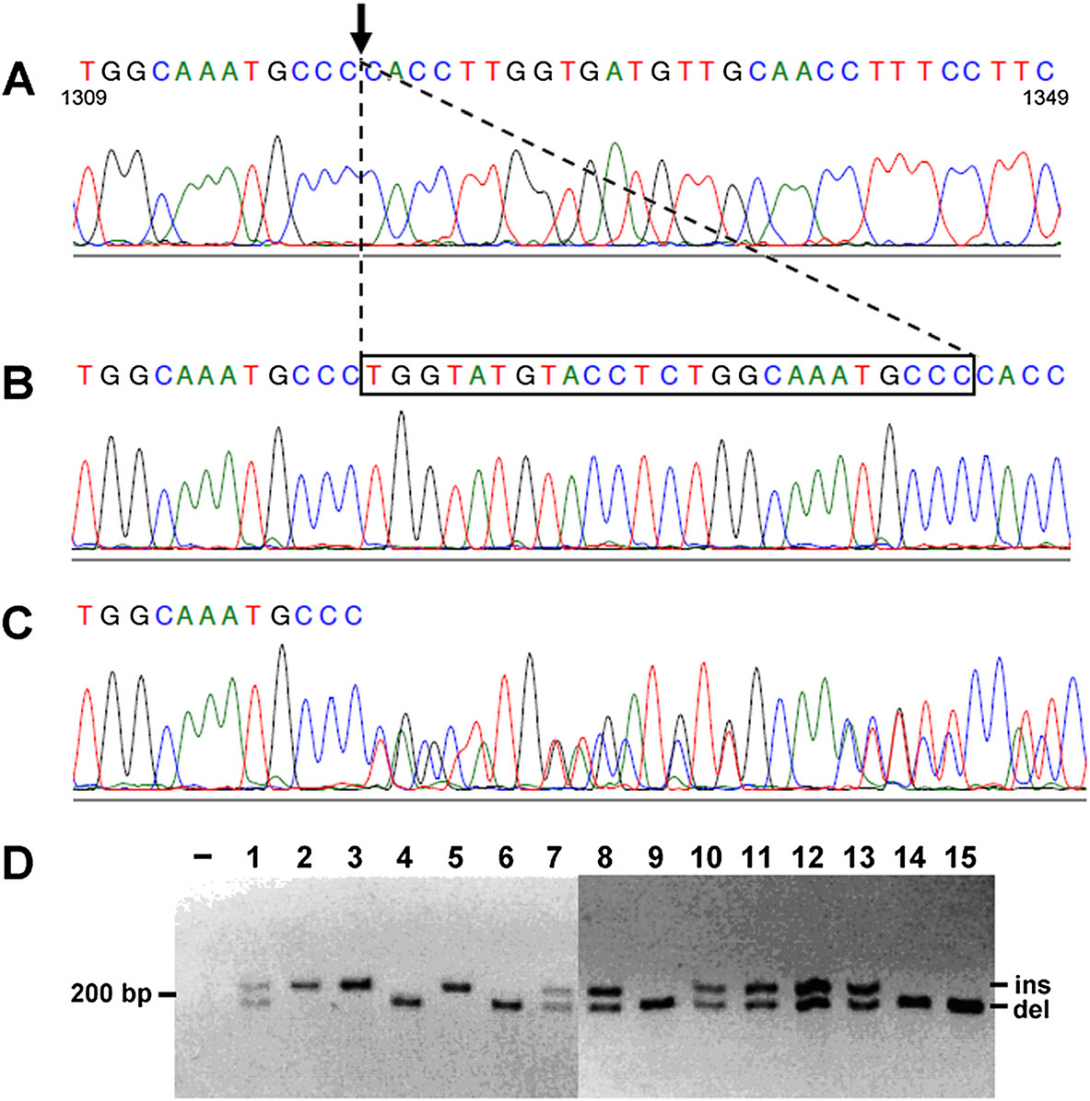
**Identification of a 25 bp insertion/deletion polymorphism in the *GJA5 *3**^**′**^**UTR.** Sequence traces from human atrial gDNA from (**A**) a deletion homozygote, (**B**) an insertion homozygote, and (**C**) a heterozygote are shown. Nucleotide sequence numbering is based on the *GJA5* Refseq entry (NM_181703). (**D**) PCR assay of atrial gDNA from 15 individuals, identifying homozygotes for the insertion (upper band, 206 bp), homozygotes for the deletion (lower band, 181 bp), and heterozygotes (with both bands). Screening of 67 atrial samples determined the minor allele frequency of the insertion to be 0.45.

We conducted a search for novel regulatory variants of *GJA5* by sequencing the proximal 1,435 bp of the *GJA5* 3^′^ UTR region in atrial tissue genomic DNA samples from 48 patients diagnosed with AF, a subset of the 67 subjects described in Table 1. We observed a novel polymorphism 22 bp downstream of the stop codon (*22G>A) in a single sample. We also observed a 25 bp insertion/deletion in multiple subjects (Figure 2A-C). The insertion/deletion, described in data uploaded by Levy and colleagues in the first reported human diploid genome sequence, is located approximately 640 bp downstream of the stop codon and approximately 1,300 bp upstream of the poly-A signal [21]. We developed a PCR length polymorphism assay to screen for the insertion/deletion in the full set of atrial gDNA derived from 67 tissue donors (Figure 2D), and we determined the minor allele (insertion) frequency to be 0.45. We also frequently observed the common 3^′^ UTR SNP rs1692141, located 53 bp downstream of the *GJA5* stop codon with a minor allele frequency of 0.237 in our samples.

### Linkage disequilibrium of the *GJA5* 3^′^ UTR insertion/deletion

**Figure 3:**
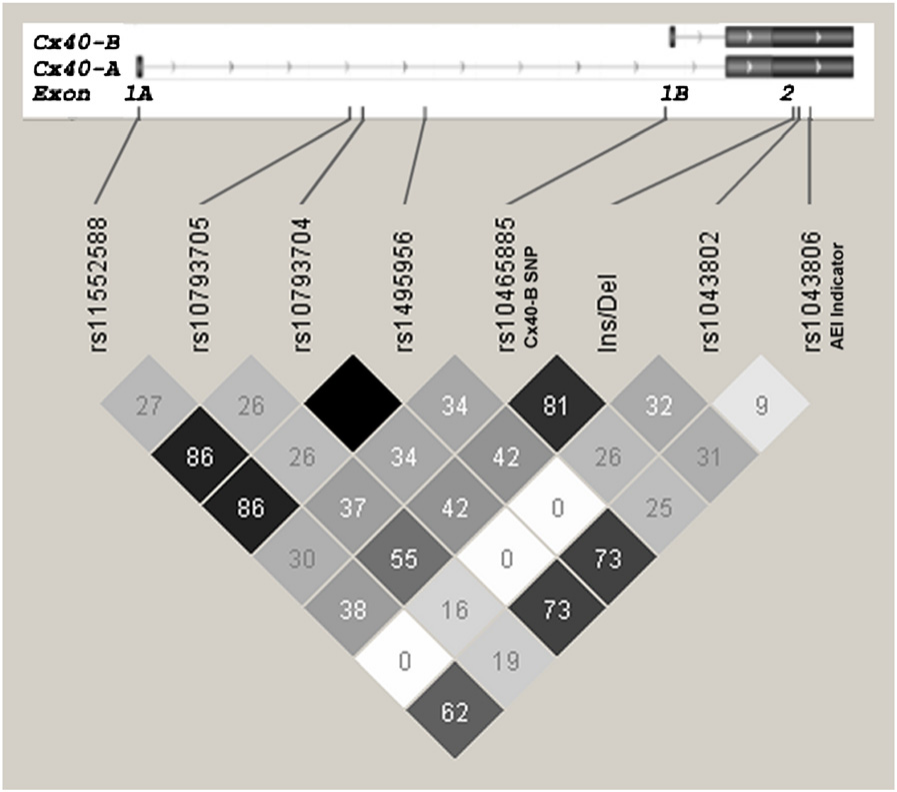
**Linkage disequilibrium of the *GJA5 *3**^**′**^**UTR insertion/deletion.** Linkage disequilibrium relationships (r^2^) between the *Cx40*-*B* promoter SNP (rs10465885), a *Cx40*-*A* promoter SNP (rs11552588), the 3^′^ UTR SNP (rs1043806) used for the allelic expression imbalance assay, the insertion deletion polymorphism (Ins/Del), and four other *GJA5* SNPs from the Illumina Hap550 BeadChip. The positions of the SNPs relative to the alternative transcripts for *Cx40*-*A* and *Cx40*-*B* are displayed. The D^′^ between rs1043806 and the insertion/deletion is 1.0 (not shown).

Haplotype analysis of the *GJA5* 3^′^ UTR insertion/deletion was conducted with the atrial tissue samples, examining eight SNP genotypes situated within the *GJA5* region. The SNP genotypes tested consisted of the common *Cx40B* promoter variant rs10465885, a *Cx40A* promoter SNP (rs11552588) and five other contiguous *GJA5* SNPs from the Illumina Hap550 BeadChip [11,22,23]. One of these SNPs, rs1043806 in the *GJA5* 3^′^ UTR, was used as the indicator SNP in an expression imbalance assay, as described below. Analysis of linkage disequilibrium (LD) relationships using Haploview v.4.1 (Broad Institute) determined that the insertion/deletion polymorphism is in partial LD with rs10465885, with r^2^ = 0.81 (Figure 3). We have previously shown that rs10465885 is modestly associated with early onset lone AF, exerting a strong cis-effect on atrial expression of the *Cx40B* transcript, and is also associated with atrial total *Cx40* mRNA levels [11]. We hypothesized therefore that the insertion/deletion polymorphism might also play a role in regulation of *Cx40* expression.

### Functional assay of the *GJA5* 3^′^ UTR insertion/deletion

**Figure 4:**
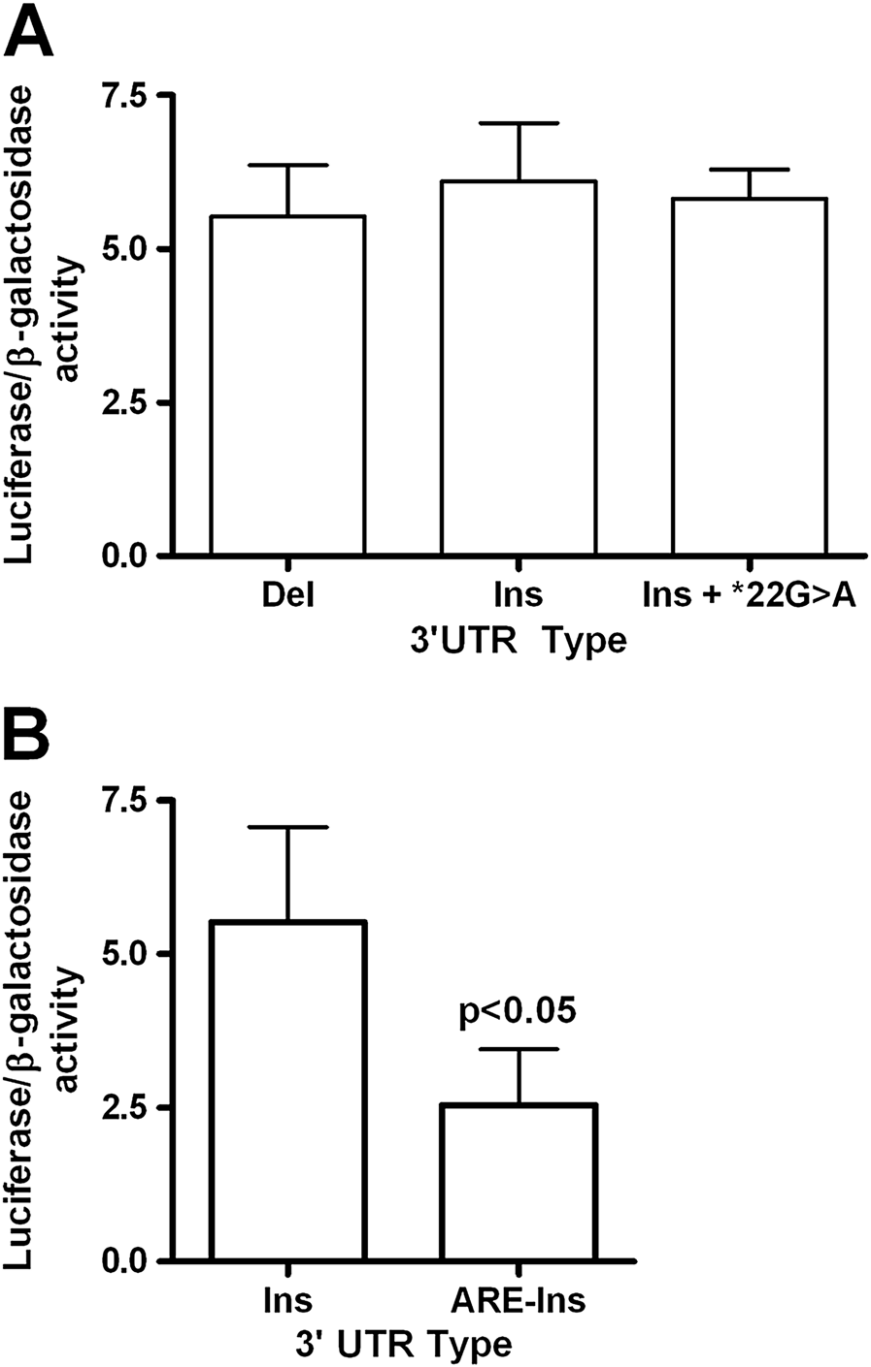
**The 3**^**′**^**UTR insertion/deletion polymorphism does not significantly affect *GJA5 *mRNA stability or translational efficiency.** (**A**) HL-1 murine cardiomyocytes transfected with vectors containing the firefly luciferase gene fused at its 3^′^ end to the *GJA5* 3^′^ UTR were measured for luciferase activity, normalized to the β-galactosidase activity of the transfection control vector. Shown are the mean activities, ± standard deviations. No significant differences in activity were observed for constructs carrying the deletion (Del, n=8), the insertion (Ins, n=4), or the insertion with a 3^′^ UTR point mutation (Ins + *22G>A, n=4) (*P* = 0.256, one-way ANOVA). (**B**) A *GJA5* 3^′^ UTR construct carrying the insertion was modified to include a mRNA instability sequence upstream of the *GJA5* 3^′^ UTR. The resulting positive control vector (ARE-Ins) demonstrated reduced luciferase activity compared to the unmodified vector (p<0.05, n=3).

We designed a luciferase reporter assay to investigate the potential effects of the common insertion/deletion on *Cx40* mRNA stability and/or translation. *GJA5* 3^′^ UTR sequences containing the deletion, the insertion, or a combination of the insertion and the 3^′^ UTR variant *22G>A were fused to the 3^′^ end of the firefly luciferase gene in a modified pcDNA3 vector [24]. The resulting insertion/deletion constructs were transiently transfected into HL-1 atrial murine cardiomyocytes and the cell lysates were assayed for luciferase activity, normalized for transfection efficiency. Comparable levels of activity were observed for cells expressing each construct (Figure 4A), suggesting that the insertion/deletion polymorphism is not associated with altered *Cx40* translational efficiency or mRNA stability. As a positive control, a luciferase reporter construct containing the *GJA5* 3^′^UTR sequence with the insertion polymorphism was modified to include an AU-rich element (ARE) that has been shown to decrease mRNA stability when present in the 3^′^UTR [13]. HL-1 murine cardiomyocytes transfected with the ARE-*GJA5* 3^′^UTR construct demonstrated a 54% reduction in luciferase activity compared to cells transfected with the unmodified reporter (Figure 4B, p<0.05).

### Allelic expression imbalance assay for the *GJA5* 3^′^ UTR insertion/deletion

**Figure 5:**
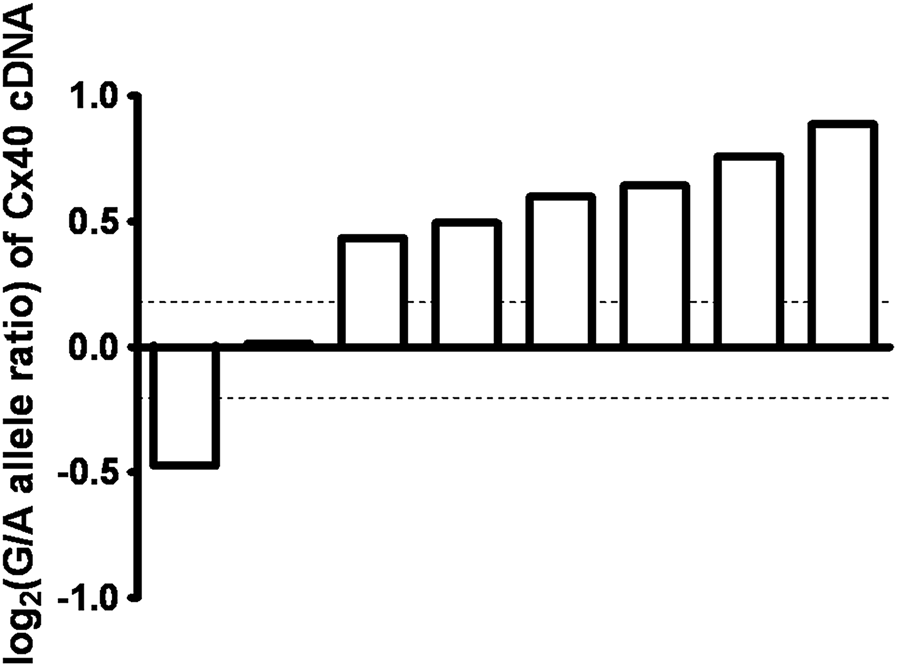
**Allelic expression imbalance analysis of *Cx40-A *mRNA.** Shown are eight atrial tissue samples heterozygous for the *GJA5* 3^′^ UTR insertion/deletion polymorphism and the indicator SNP rs1043806. Each bar represents the allelic expression ratio for an individual subject, based on peak heights for cDNA/gDNA at the indicator SNP site. Significant allelic mRNA imbalance is found when the cDNA allelic ratio falls outside of a 95% confidence interval created by the gDNA allelic ratios (dotted lines). The G allele of the indicator SNP was always found together with the deletion allele of the insertion/deletion polymorphism. Six samples demonstrated weak allelic expression imbalance of *Cx40* favoring the deletion-G allele. One sample was not significantly associated with allelic expression imbalance and another sample exhibited *Cx40* expression imbalance in the opposite direction.

The potential effect of the insertion/deletion on atrial *Cx40* mRNA levels was also tested using a previously described allelic expression imbalance assay [11,16]. In this assay, the relative expression of two allelic *Cx40* transcripts was quantified by direct cDNA sequencing in subjects heterozygous for a common indicator SNP contained within the mRNA. PeakPicker 2 software [15,25] was used to calculate the allelic ratios by measuring peak heights from cDNA sequence traces, normalized to the allelic ratio of the corresponding heterozygous gDNA. Of the two alternatively spliced *Cx40* transcripts, *Cx40A* mRNA expression was measured in order to exclude the strong allelic imbalance observed for the *Cx40B* transcript in rs10465885 heterozygotes. We tested eight atrial tissue samples heterozygous for the insertion/deletion and the indicator SNP rs1043806. The minor G allele of rs1043806 is in partial LD with the insertion/deletion (r^2^ = 0.31, Figure 3) and is consistently in phase with the deletion allele (D' = 1.0). Six of the eight samples demonstrated weak allelic expression imbalance, yielding a mean 1.56-fold increase in *Cx40* expression for the deletion-G allele (Figure 5). The remaining two samples showed no change in *Cx40* levels or imbalance in the opposite direction, respectively (Figure 5), yielding an overall mean 1.39-fold increase for the deletion-G allele. The observed pattern of incomplete expression imbalance indicates that the insertion/deletion is not directly associated with regulation of *Cx40* expression; however, the insertion/deletion polymorphism may be in partial LD with a separate unidentified modest-strength regulatory variant.

### Statistical analyses of Cx40 missense allele frequencies in human blood and atrial tissue

Fisher’s exact probability test was used to compare the frequencies of nonsynonymous variant *Cx40* alleles observed in our studies with those reported by groups studying similar sample cohorts. We found no statistically significant difference (p = 0.56) between the frequency of missense *Cx40* alleles in our 91 lone AF blood samples (variant:non-variant alleles = 0:182, 0%) and the frequency observed in 218 lone AF blood samples by Yang and colleagues (3:433, 0.69%) [26]. However, we observed a significant difference (p = 0.03) when comparing *Cx40* missense allele frequencies in our 34 lone AF tissues (average age at surgery 54 years), in which we detected a single nonsynonymous variant (variant:non-variant alleles = 1:67, 1.47%), and the frequency observed by Gollob *et al*. in 15 highly selected and young (average age of onset 45 years) lone AF tissue donors, in which four novel heterozygous *Cx40* missense mutations were found (4:26, 13.3%) [9].

## Discussion

We conducted direct sequencing of the *GJA5* coding region in 91 lone AF blood samples and 67 atrial tissue samples (34 lone AF, 30 mixed AF, and 3 donors) from individuals of predominantly European ancestry, identifying a novel nonsynonymous SNP (339G>C), a novel synonymous SNP (951T>C), and a previously reported synonymous SNP (rs2232191). In the 3^′^ UTR we identified a single novel variant (*22G>A). We also determined that a 25 bp insertion/deletion polymorphism in the *GJA5* 3^′^ UTR is common and in partial LD with the *Cx40* transcript B promoter SNP rs10465885, which is associated with early onset lone AF and strongly correlated with *Cx40B* and total *Cx40* expression [11]. However, reporter gene transfection and *Cx40A* allelic expression imbalance assays indicated that the insertion/deletion is not directly associated with changes in *Cx40* expression. The modest and inconsistent 1.39-fold effect observed for the insertion/deletion in the 8 subjects assayed for *Cx40A* allelic expression imbalance was much weaker than the consistent 3.3-fold effect that rs10465885 has on allelic expression imbalance of the *Cx40B* transcript [11]. These allelic expression imbalance results support the hypothesis that the insertion/deletion is in partial LD with a separate modest regulatory variant that is responsible for the partial allelic imbalance observed.

The most recent and largest AF genome-wide association study has not identified common variants in or near the *GJA5* gene to be associated with AF at the conservative genome-wide significance threshold [27]. However, we have previously identified a common *GJA5* promoter variant that both alters *Cx40* expression levels and is weakly associated with AF [11]. Since not all of the heritability for common traits can be attributed to common variants, sequencing studies can be used to identify rare variants that may contribute to AF susceptibility. Recent sequencing studies screening lone AF patients for novel *Cx40* coding variants have observed very different rare allele frequencies. In fifteen young, idiopathic AF patients of Western European descent, Gollob *et al*. identified one germ line and three somatic nonsynonymous variants in the *Cx40* coding region using DNA derived from atrial tissue samples and blood [9]. In contrast, we observed only one nonsynonymous variant in our 34 lone AF tissues. We hypothesize that this difference may be due to the high degree of selection of lone AF subjects in the cohort studied by Gollob. In a prior analysis of *Cx40* genetic variants using blood-derived DNA, Yang and colleagues reported the discovery of three nonsynonymous *Cx40* variants in 218 lone AF patients of Chinese descent, and no novel variants in 200 control subjects [26]. Our studies found no nonsynonymous *Cx40* variants in blood-derived DNA samples from 91 lone AF subjects, which was not statistically different from the frequency observed by Yang. Combined with our finding of no *Cx40* nonsynonymous variants in 30 mixed AF atrial tissue samples, these results suggest that *Cx40* coding SNPs are uncommon in lone and secondary AF patient populations.

The current study has several limitations. First, all samples were predominantly derived from patients of European ancestry and the sample size was limited to 91 lone AF blood-derived DNA samples and 67 atrial tissue samples, of which only 34 were lone AF subjects. Second, we did not possess paired blood and tissue samples, and thus were unable to determine whether the rare *GJA5* variants discovered in atrial tissue were of germline or somatic origin. We also did not have a large cohort of donor atrial tissue available that could be used to compare the frequency of rare variants in AF vs. control atria. Finally, our transfection study was performed in HL-1 cells, an atrial-derived, immortalized mouse cell line that demonstrates some atrial-like properties [12]. It is therefore possible that the Cx40 3^′^UTR insertion/deletion polymorphism, which we observed to have no effect on reporter activity, could still modify gene expression *in vivo* in the human atrium.

## Conclusions

In 158 atria- and blood-derived DNA samples from subjects with AF, we observed only one nonsynonymous rare genetic variant in the *GJA5* gene. We determined that a 25 bp insertion-deletion polymorphism is common in the *GJA5* 3^′^ UTR, but we could detect no functional role for this variation. Our study suggests that aggregations of different rare nonsynonymous *GJA5* genetic variants are not commonly observed among AF patients. Although rare, *GJA5* variants that alter Cx40 function may be directly causal of AF.

## Abbreviations

Cx40: Connexin-40; AF: Atrial fibrillation; SNP: Single Nucleotide Polymorphism; LD: Linkage Disequilibrium.

## Competing interests

The authors declare that they have no competing interests.

## Authors’ contributions

GDT conducted direct sequencing of the GJA5 coding and proximal 3^′^ UTR regions, allelic expression imbalance analysis, statistical analysis of Cx40 missense allele frequencies, and drafted the manuscript. RCW carried out sequencing of the GJA5 3^′^ UTR and characterization of the insertion/deletion polymorphism, including the PCR screen, haplotype analysis and functional assays, and assisted in drafting the manuscript. DRW participated in the design of the study and provided blood and atrial tissue samples. MKC participated in the design of the study, performed chart reviews and provided clinical characteristics of patient samples. JDS conceived of the study, participated in its design and coordination and helped to draft the manuscript. All authors read and approved the final manuscript.

### **Funding sources**

This work was supported by NIH/ National Heart, Lung and Blood Institute grant HL090620 and the supplementary American Recovery and Reinvestment Act (ARRA) grant HL090620-01A1S2 to Drs. Chung, Barnard, Smith, and Van Wagoner. It was also supported in part by NIH/National Center for Research Resources (NCRR) Case Western Reserve University/Cleveland Clinic CTSA UL1-RR024989 (Chung, Van Wagoner); the Heart and Vascular Institute, Department of Cardiovascular Medicine, Cleveland Clinic (Chung); Leducq Foundation grant 07-CVD 03 (Van Wagoner, Chung); and the Atrial Fibrillation Innovation Center, State of Ohio (Van Wagoner, Chung). Robert Wirka was a Howard Hughes Medical Institute Medical Research Training Fellow. Dr. Tchou was partially supported by a Case Western Reserve University training grant in Cardiovascular Research from the NIH (T32HL1053381).

## Pre-publication history

The pre-publication history for this paper can be accessed here:

http://www.biomedcentral.com/1471-2350/13/102/prepub
